# Omega 3 Fatty Acids Intake Does Not Decrease the Risk of Rheumatoid Arthritis Occurrence: A Meta-Analysis. Comment on Tański et al. The Relationship between Fatty Acids and the Development, Course and Treatment of Rheumatoid Arthritis. *Nutrients* 2022, *14*, 1030

**DOI:** 10.3390/nu15030539

**Published:** 2023-01-20

**Authors:** Sylvain Mathieu, Bruno Pereira, Claire Daïen, Anne Tournadre, Martin Soubrier

**Affiliations:** 1 Faculty of Medicine, Université Clermont Auvergne, Inserm, Neuro-Dol, 63000 Clermont-Ferrand, France; 2 Département de Rhumatologie, CHU Gabriel Montpied, Université Clermont Auvergne, 63000 Clermont-Ferrand, France; 3 Rheumatology Department, Gabriel Montpied Teaching Hospital, 58 Rue Montalembert, 63003 Clermont-Ferrand, France; 4 Département de Recherche Clinique et Innovation, CHU Gabriel Montpied, 63000 Clermont-Ferrand, France; 5 Département de Rhumatologie, CHU Montpellier, Université de Montpellier, 34295 Montpellier, France; 6 INSERM U1046–CNRS UMR 9214 PHYMEDEXP, Université de Montpellier, 34295 Montpellier, France

In an article published in *Nutrients*, Tański et al. performed a systematic review and concluded that omega-3 fatty acids might contribute to a reduced incidence of rheumatoid arthritis (RA) [1]. We wish to add further data to these conclusions. We performed a meta-analysis to investigate the risk of RA occurrence in patients consuming omega-3 fatty acids.

We searched MEDLINE, EMBASE, and The Cochrane Library to identify all reports of interest that were published prior to 13 October 2022/using the search terms: “(rheumatoid OR arthritis OR joint OR articular) AND omega”. The studied population comprised patients with RA; the intervention analyzed was the oral supplementation of omega-3; the controls were patients receiving a placebo, and the outcome retained was the occurrence of RA cases. We selected articles published in English or French and retrieved a total of 2239 articles. The incidence of RA occurrence in omega-3 users and non-users was calculated by a meta-analysis of proportions which were estimated using the inverse-variance method. The Mantel–Haenszel procedure was used to determine the odds ratio (OR).

The database research found seven studies to which eight references were added by reading the article references [2,3,4,5,6,7,8,9,10,11,12,13,14,15,16]. In the study of Hu et al., two cohorts were described [6] from the Nurses’ Health Study. We separately analyzed these two cohorts, which is why the study of Hu et al. appeared twice as “Hu 2015” and “Hu 2015 bis”. Four studies were excluded because data were not usable for meta-analysis [13,14] or because the studies included omega-3 non-users [15,16]. Therefore, we finally included 11 articles, i.e., 12 studies involving a total of 396,388 patients consuming omega-3 via fish intake (*n* = 10) or fish oil intake (*n* = 1) and 105,686 omega-3 non-users (Figure 1). Five studies were the case–control, and seven were cohorts. In the 12 studies, 6918 RA were reported in omega-3 users with an incidence of 9.2% [6.4, 12.6%]; conversely, in omega-3 non-users, 1960 RA were reported with an incidence of 11.1% [10.5, 11.8%]. There were no differences between the groups in the incidence of RA depending on the type of study (case–control or cohort studies). The overall meta-analysis showed no difference in the risk of RA occurrence depending on the omega-3 consumption (OR = 0.98 [0.87, 1.11]) (Figure 2). The seven cohort studies revealed no difference in the risk of RA occurrence depending on omega-3 consumption (Figure 3). Conversely, the five case–control studies found a decrease in RA risk among omega-3 users. 

Our results are rather in favor that omega-3 intake is not associated with a decreased risk of RA occurrence, which is different from the conclusion of Tański et al. and is more concordant with the results of Hanh et al. that were not included in the Tanski review. 

## Figures and Tables

**Figure 1:**
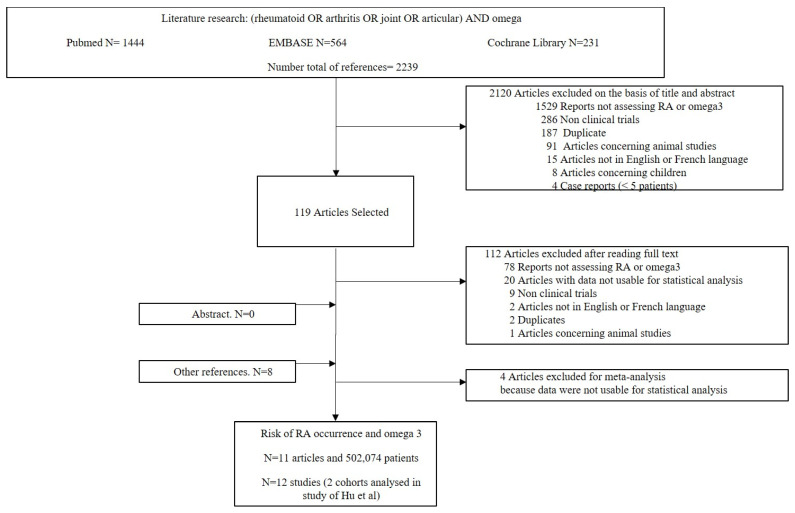
Flowchart of study selection.

**Figure 2:**
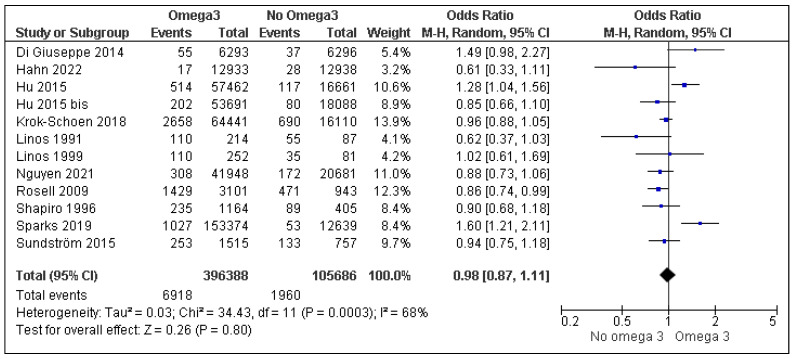
Forest plot of the risk of RA occurrence between omega 3 users and non-users [2,3,4,5,6,7,8,9,10,11,12].

**Figure 3:**
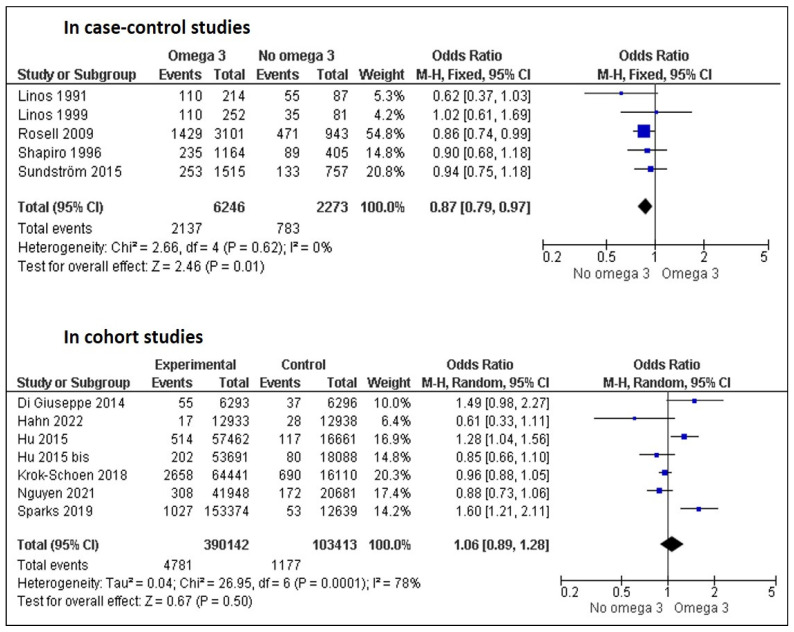
Forest plot of the risk of RA occurrence between omega 3 users and non-users depending on the type of studies [2,3,4,5,6,7,8,9,10,11,12].

## References

[1] Tański W., Swiatoniowska-Lonc N., Tabin M., Jankowska-Polanska B. (2022). The relationship between fatty acids and the development, course and treatment of rheumatoid arthritis. Nutrients.

[2] Hahn J., Cook N., Alexander E., Friedman S., Walter J., Bubes V. (2022). Vitamin D and marine omega 3 fatty acid supplementation and incident autoimmune disease: VITAL randomized controlled trail. BMJ.

[3] Linos A., Kaklamanis E., Kontomerkos A., Koumantaki Y., Gazi S., Vaiopoulos G. (1991). The Effect of Olive Oil and Fish Consumption on Rheumatoid Arthritis-A Case Control Study. Scand. J. Rheumatol..

[4] Shapiro J., Koepsell T., Voigt L., Dugowson C., Kestin M., Nelson J. (1996). Diet and rheumatoid arthritis in women: A possible protective effect of fish consumption. Epidemiology.

[5] Di Giuseppe D., Wallin A., Bottai M., Askling J., Wolk A. (2014). Long-term intake of dietary long-chain n-3 polyunsaturated fatty acids and risk of rheumatoid arthritis: A prospective cohort study of women. Ann. Rheum. Dis..

[6] Hu Y., Costenbader K., Gao X., Hu F., Karlson E., Lu B. (2015). Mediterranean diet and incidence of rheumatoid arthritis in women. Arthritis Care Res..

[7] Sparks J., O’Reilly É., Barbhaiya M., Tedeschi S., Malspeis S., Lu B. (2019). Association of fish intake and smoking with risk of rheumatoid arthritis and age of onset: A prospective cohort study. BMC Musculoskelet. Disord..

[8] Rosell M., Wesley A., Rydin K., Klareskog L., Alfredsson L., the EIRA study group (2009). Dietary fish and fish oil and the risk of rheumatoid arthritis. Epidemiology.

[9] Krok-Schoen J., Brasky T., Hunt R., Rohan T., Baker T., Li W. (2018). Dietary long-chain omega-3 fatty acid intake and arthritis risk in the Women’s Health Initiative. J. Acad. Nutr. Diet..

[10] Sundström B., Johansson I., Rantapää-Dahlqvist S. (2015). Diet and alcohol as risk factors for rheumatoid arthritis: A nested case–control study. Rheumatol. Int..

[11] Linos A., Kaklamani V., Kaklamani E., Koumantaki Y., Giziaki E., Papazoglou S. (1999). Dietary factors in relation to rheumatoid arthritis: A role for olive oil and cooked vegetables?. Am. J. Clin. Nutr..

[12] Nguyen Y., Salliot C., Mariette X., Boutron-Ruault M.C., Seror R. (2022). Fish consumption and risk of rheumatoid arthritis: Findings from the E3N cohort study. Nutrients.

[13] Pedersen M., Stripp C., Klarlund M., Olsen S., Tjønneland A., Frisch M. (2005). Diet and risk of rheumatoid arthritis in a prospective cohort. J. Rheumatol..

[14] Benito-Garcia E., Feskanich D., Hu F., Mandl L., Karlson E. (2007). Protein, iron, and meat consumption and risk for rheumatoid arthritis: A prospective cohort study. Arthritis Res. Ther..

[15] Nguyen H.D., Oh H., Kim M.S. (2022). An increased intake of nutrients, fruits and green vegetables was negatively related to the risk of arthritis and osteoarthritis development in the aging population. Nutr. Res..

[16] Nguyen H.D., Oh H., Kim M.S. (2022). Higher intakes of nutrients are linked with a lower risk of cardiovascular diseases, type 2 diabetes mellitus, arthritis, and depression among Korean adults. Nutr. Res..

